# Natural infection of synathropic rodent species *Mus musculus* and *Rattus norvegicus* by *Leishmania infantum* in Sesimbra and Sintra – Portugal

**DOI:** 10.1186/1756-3305-6-88

**Published:** 2013-04-08

**Authors:** Marcos Helhazar, José Leitão, Ana Duarte, Luís Tavares, Isabel Pereira da Fonseca

**Affiliations:** 1 CIISA, Fac Med Vet, Av, Universidade Técnica, 1300-477, Lisboa, Portugal; 2 Centro Médico Veterinário de Cabra Figa, Rua da Liberdade, lote 889-A Cabra Figa, 2635-128, Rio de Mouro, Portugal

**Keywords:** Leishmaniosis, *Leishmania infantum*, Rodents, *Mus musculus*, *Rattus norvegicus*, Reservoir

## Abstract

**Background:**

Canine leishmaniosis caused by *Leishmania infantum* is a parasitic zoonotic disease transmitted by phlebotomine sandflies (Diptera: Psychodidae). Genus *Phlebotomus* is the biological vector in the Old World and *Lutzomyia* in the New World. The dog is the domestic reservoir host but other animals like the fox (*Vulpes vulpes*) and rodents are known to maintain the infection in both sylvatic and domestic cycles.

**Methods:**

To identify the role of synanthropic rodents *Mus musculus* and *Rattus norvegicus* as reservoir hosts for *Leishmania infantum* natural infection, 30 rodents were captured under a trap rodent control program in two private dog shelters from Sintra and Sesimbra, located in the Lisbon Metropolitan Area, known to be endemic for canine leishmaniosis in Portugal. Tissue samples were screened for the presence of *Leishmania* amastigotes by qPCR and parasitological analysis.

**Results:**

A total of 33.3% (9/27) of *Mus musculus* rodents revealed the presence of *Leishmania* spp. DNA while 29.6% (8/27) were positive in the parasitological analysis. Concerning *Rattus norvegicus* (n=3), one animal revealed infection only by parasitological analysis.

**Conclusions:**

Our results identified for the first time in Portugal the presence of *Leishmania* infection in both rodent species. As susceptible hosts, infected *Mus musculus* and *Rattus norvegicus* may increase the risk for dog and human infection in households and surrounding areas, enhancing the need for efficient rodent control measures in shelters and risk zones to prevent transmission of the infection.

## Background

In Europe, visceral zoonotic leishmaniosis is a protozoan disease caused by *Leishmania infantum*. The parasite is transmitted by female infected sandflies from the genus *Phlebotomus* during feeding. The domestic dog is the main reservoir host for *Leishmania infantum* but other reservoirs are known, such as wild carnivores and rodents [1-3]. In Portugal, canine leishmaniosis is an endemic disease with an infection rate of 6.31% obtained during a serological survey performed in 3.974 dogs from veterinary clinics in Mainland Portugal [4]. Recently, the domestic cat has also been suggested as a reservoir host and in Northern Portugal, a 2.8% seroprevalence was found in 316 domestic cats [5].

Man can be infected by *Leishmania infantum*, especially in cases of immunodeficiency caused by diseases such as AIDS or other conditions [6]. In Portugal, between 2000 and 2009, 173 cases of human visceral leishmaniosis were diagnosed. The emergence of 15 to 20 new cases is estimated yearly [7].

The disease is transmitted by *Phlebotomus perniciosus* and *Phlebotomus ariasi*, both sandfly vectors reported in Portugal [7]. Alternative ways of transmission such as blood transfusion, vertical and venereal transmissions have also been inferred [8].

The identification of natural hosts of *Leishmania* is fundamental to better understand the epidemiology of the disease. Due to close proximity between rodents, dogs and humans, the importance of rodents as reservoir hosts for different *Leishmania* species have already been described worldwide [3]. In Brazil, several authors reported infected rodents in natural conditions with *Leishmania* spp. [9-11] and *L. donovani* complex, *L. mexicana* complex and *L. braziliensis* complex [12]. The same occurred in Venezuela with identification of *L. donovani* complex [13,14] and *Leishmania* sp. [7]. In Mexico, *Leishmania* spp. were reported [15]. In Italy, *L. infantum*, was identified [16], as well as in Cyprus [17] and Greece [18]. In Iran, *L*. *major*, *L*. *turanica*, *L*. *donovani*, *L*. *infantum*, *L*. *major* and *L*. *gerbilli* were also detected [19-23].

In Portugal, to our knowledge no data is available concerning natural *Leishmania* infection in any rodent species. The aim of this work was to investigate the role of *Mus musculus and Rattus norvegicus* rodents as natural reservoirs for *Leishmania* spp. in Sintra and Sesimbra, both canine leishmaniosis endemic areas in Central Portugal.

## Methods

The private dog shelters, located in two rural areas of Sintra and Sesimbra (Central Portugal), were surrounded by abundant vegetation and trees. The shelter from Sintra, with 215 dogs under its care, was surrounded by walls about three meters high. The kennels were built with brick walls and tile ceilings. The shelter from Sesimbra, with a population of 230 dogs, was surrounded by grating two meters high and the ground was covered with cement. The kennels were built with brick walls and fiber cement ceilings, also with grating doors. The prevalence of canine leishmaniosis was 2.3% (5/215) and 5.2% (12/230), respectively (data not published).

Thirty rodents (27 *Mus musculus* and 3 *Rattus norvegicus*) were collected between May and October 2011 under a trap rodent control program applied in each private shelter. No ethical approval for animal trapping is officially required since these rodents are considered pest species. Dead specimens of *Mus musculus* collected from Sesimbra and *Rattus norvegicus* collected from Sintra were kept refrigerated for a maximum period of 6 hours before being transported to the Faculdade de Medicina Veterinária - Universidade Técnica de Lisboa (FMV/UTL) and processed by standard necropsy procedure.

The rodent’s genus and species were determined by external characteristics including color, body length and ear lobes, tail, feet, teeth and cranium measurements [24].

For each rodent, liver and spleen fragments were collected and stored in 10% formaldehyde and in RNAlater®. Imprint smears were performed from the liver and spleen. Fragments of both ear lobes were collected for RNAlater®, along with tail lesions whenever observed.

Liver and spleen imprint smears were fixed with methanol for 60 seconds, stained with 10% Giemsa for 60 seconds and observed under 1000× magnification in an optical microscope.

Formaldehyde fixed tissues were later processed routinely for paraffin embedding, 3 μm sections were cut, stained with hematoxylin and eosin and observed under an optical microscope using a 400× and 1000× magnification.

Tissue samples from spleen, liver, tail and ear lobe, were sliced in 10–20 mg fragments and processed for total DNA extraction using DNeasy Blood & Tissue Kit ® (QIAGEN, Germany) following the manufacturer’s instructions. Detection of *Leishmania* spp. nucleic acid was performed by real time PCR (qPCR) using the Applied Biosystems® 7300 Real-time thermocycler. A set of primers and TaqMan® probe were calculated by the NCBI primer blast tool, available through http://www.ncbi.nlm.nih.gov/tools/primer-blast/, based on the *Leishmania* sp. small circle kinetoplast nucleotide sequence, generating a forward primer 5´-AGGTGTCGTAAATTCTGGAA-3´, a reverse primer 3´-CGGGATTTCTGCACCATT and a Taqman® probe FAM 5´- AATTCCAAACTTTTCTGGTCCTCCGGGTAG TAMRA – 3´, spawning a 124 bp product.

The qPCR amplification was performed in a 20 μl reaction volume with 2× TaqMan® Gene Expression Master Mix (Applied Biosystems), 3 μM of primer forward, 3 μM of primer reverse, 2.5 μM of Taqman® probe and 50 ng of total DNA. Cycling conditions included an initial denaturation step at 95°C for 10 minutes followed by 50 cycles at 95°C for 15 seconds and 60°C for 1 minute.

The assay specificity was confirmed by sequencing of the amplicon after plasmid cloning (pGEM®, Promega), according to the manufacturer´s instructions. To estimate the parasitic load serial tenfold dilutions of the recombinant plasmid DNA were used to generate a standard curve with a r2=0.997 and a slope of −3.6 to −3.4, with the 7300 System SDS software® (Applied Biosystems), corresponding to a reaction efficiency of 90% - 100%.

The assay sensitivity surpassed the detection of 10 target copies. For parasitic load quantification, only samples amplified within the Ct range of the standard curve were considered.

## Results

### External examination

In the external examination of the thirty rodents the body condition was normal, according to the species parameters. Two rodents revealed eroded tail lesions. One was detected in a *Mus musculus* and another in a *Rattus norvegicus*. The lesions measured 4 mm and 8 mm of diameter, respectively.

### Parasitological analysis

**Figure 1:**
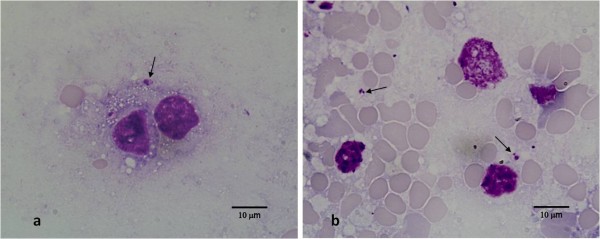
***Leishmania *amastigotes (arrows) in liver smear (a) and spleen smear (b) of *Mus musculus*.**

*Leishmania* amastigotes (Figure 1) were detected in 8 *Mus musculus* (n=27); 2 animals were positive in both the liver smear and in the spleen histological section; 2 animals were positive in both liver and spleen smears; 3 animals were positive in the spleen histological section and 1 animal was positive in the liver smear. Concerning *Rattus norvegicus* (n=3), one animal was positive in the liver and in the spleen smears simultaneously.

All positive samples showed no more than three amastigotes per microscopic field.

### Molecular analysis

*Leishmania* kDNA was detected in 9 ear lobe skin samples from *Mus musculus.* The parasitic load ranged between the detection of residual values to 393 targeted copies, indicative of low parasite burden, as already detected in the parasitological analysis (Table 1).

**Table 1:** Results obtained from molecular and parasitological analysis

**Rodent species**	**Rodent nº**	**qPCR analysis (parasitic load)**			**Parasitological analysis**			
		Ear lobe	Liver	Spleen	Liver smear	Liver H.S.	Spleen smear	Spleen H.S.
*Rattus norvegicus*	1	N	N	N	N	N	N	N
	2	N	N	N	N	N	N	N
	3	N	N	N	**P**	N	**P**	N
*Mus musculus*	4	N	N	N	**P**	N	**P**	N
	5	**P** (72)	N	N	N	N	N	**P**
	6	**P** (50)	N	N	N	N	N	N
	7	N	N	N	N	N	N	N
	8	N	N	N	**P**	N	N	N
	9	N	N	N	N	N	N	N
	10	**P** (100)	N	N	N	N	N	**P**
	11	N	N	N	N	N	N	N
	12	N	N	N	N	N	N	N
	13	**P** (393)	N	N	N	N	N	N
	14	N	N	N	N	N	N	N
	15	N	N	N	N	N	N	N
	16	N	N	N	N	N	N	N
	17	N	N	N	**P**	N	N	**P**
	18	N	N	N	N	N	N	N
	19	**P** (50)	N	N	**P**	N	**P**	N
	20	N	N	N	N	N	N	N
	21	N	N	N	N	N	N	N
	22	N	N	N	N	N	N	N
	23	N	N	N	N	N	N	N
	24	**P** (resid)	N	N	N	N	N	N
	25	N	N	N	N	N	N	N
	26	N	N	N	N	N	N	N
	27	**P** (resid)	N	N	N	N	N	**P**
	28	N	N	N	N	N	N	N
	29	**P** (resid)	N	N	N	N	N	N
	30	**P** (resid)	N	N	**P**	N	N	**P**

Legend: **P**: Positive result; N: Negative result; H.S.: Histological section; resid: residual.

The tail skin lesions were negative to *Leishmania* kDNA, as were the liver and spleen samples from *Mus musculus* and *Rattus norvegicus*.

## Discussion

Despite the possible importance of the rodent´s role as reservoir hosts for canine and human leishmaniosis, to our knowledge, no previous studies on *Leishmania* infection in any rodent species had been published in Portugal.

Our results revealed the presence of *Leishmania* amastigotes in eight *Mus musculus* and in one *Rattus norvegicus* visceral sample. Despite *Leishmania* amastigote detection in the liver and spleen, no positive results were obtained by qPCR from these samples. Regarding nucleic acid detection, positive samples were obtained from the skin but not from the liver and spleen suggesting a cutaneous *Leishmania* infection. Considering the high sensitivity of the qPCR assay visceral negative results may be due to random selected fragments for amplification and microscopic observation.

In experimental studies, early dissemination of *Leishmania* parasites occurs in the spleen [18]. In susceptible mice, the spleen constitutes the organ where the adaptive immune response to the parasite mainly takes place. In this study, in spite of systemic dissemination being present on 6 rodent’s livers (on parasitological analysis) and 8 rodent´s spleens (3 on parasitological analysis and 5 on histopathological analysis), no positive results were obtained by qPCR on spleen samples. Similar PCR results were also described by other authors working with specimens of *Mus musculus* infected with *Leishmania major*[23].

In the molecular assay, 9 ear lobe skin samples from *Mus musculus* (n=27) were positive to *Leishmania* DNA. Although no DNA sequencing was performed, the use of a specific Taqman® probe to *Leishmania infantum* in the qPCR strongly suggests the detection of this species in our samples. Furthermore, *Leishmania infantum* is known to be the only *Leishmania* species causing zoonotic visceral leishmaniosis in Portugal and in the Mediterranean countries [25].

In our study, an infection rate of 33.3% (9/27) was found in ear lobe skin of *Mus musculus*. This result is higher than the ones obtained in Brazil with 13.7% (3/22) [10] and Iran with 19% (4/21) [23]. This occurrence may have an important epidemiological role on the transmission of the parasite.

The identification of positive ear lobe skin samples in our *Mus musculus* rodents indicates the parasite cutaneous availability to *Phlebotomus* species, allowing the infection to spread during sandfly feeding, not only between rodents but also to other hosts. In fact, in an experimental study, using specimens of *Rattus rattus* and *Leishmania tropica*, the ear lobe was suggested as a preferential feeding spot for sandflies, probably due to thinner skin, easy access and abundant blood flow. In addition, the parasite DNA persisted in the ear lobe for 24 months post-inoculation and the ability to transmit the infection to sandflies from month 1 to month 24 post-inoculation was observed [26]. Although the parasite number in the blood was not assessed in this work, considering that the parasite load ranged between 50 to 393 copies in 5 animals, it is safe to conclude that the parasites were present in the skin in sufficient numbers to infect sandflies. Unfortunately, in this work, it was not possible to perform specific antibody titers to correlate data with the obtained molecular results.

This work reports for the first time in Portugal, the natural infection of *Leishmania infantum* in *Mus musculus* and *Rattus norvegicus*, suggesting these animals probably have a role as reservoirs in the *Leishmania infantum* life cycle. *Mus musculus* and *Rattus norvegicus* are extremely prolific animals, with a 12 and 24 months life expectancy respectively, maintaining the parasite availability at least for one year, even during low sandfly activity season. The infection rate obtained by qPCR on *Mus musculus* rodents (33.3%) strongly suggests the occurrence of contact with infected sandflies, especially considering the preferential nightfall activity period overlap. Also, the absence of skin lesions strongly suggests a non-pathogenic infection course, as already described in Brazil [10,11] and in Iran [22,23].

This study was performed in a peridomestic environment in two regions, with a canine leishmaniosis prevalence of 2.3% (5/215) in Sintra and 5.2% (12/230) in Sesimbra. *Mus musculus* and *Rattus norvegicus* were found to be naturally infected by *Leishmania infantum* with an overall prevalence of 43.3% (13/30)*.* These species are known to inhabit in close proximity to human housing, especially where shelter and food are provided like those found in Sesimbra and Sintra. This process of domiciliation may be important in the urban and periurban cycle of *Leishmania infantum*. Further studies are necessary to evaluate the ability of these rodents to effectively infect vector sandflies in order to establish *Mus musculus* and *Rattus norvegicus* as reservoir hosts for *Leishmania infantum*.

As susceptible hosts with a potential role in the epidemiology of this zoonotic disease, infected rodents may increase the risk for dog and human infection in households and surrounding areas, enhancing the need for an efficient rodent control measure in shelters and risk zones to prevent the transmission.

## Conclusions

This work reports for the first time in Portugal, the natural infection of *Leishmania infantum* in *Mus musculus* and *Rattus norvegicus*, implicating these animals as possible reservoirs in the *Leishmania infantum* life cycle, according to the established criteria by WHO [6]. As susceptible hosts with a potential role in the epidemiology of this zoonotic disease, infected rodents may increase the risk for dog and human infection in households and surrounding areas, enhancing the need for efficient rodent control measures in shelters and risk zones to prevent the transmission of infection.

## Competing interests

Authors declare that they have no competing interests whatsoever.

## Authors’ contributions

MH - Study design support, parasitological studies carried out, research and draft of the manuscript JPL - Study conception, design and coordination, parasitological work support, research and manuscript draft support. AD - Molecular studies carried out, data interpretation and manuscript draft support. LT - Molecular studies work out, data interpretation and manuscript draft support IPF - Study design and coordination, data interpretation, and manuscript draft support. All authors read and approved the final version of the manuscript.

## References

[B1] Ashford RW Leishmaniasis reservoirs and their significance in control Clin Dermatol 1996 14 523 532 10.1016/0738-081X(96)00041-7 8889331

[B2] Ashford RW The leishmaniasis as emerging and reemerging zoonosis Int J Parasitol 2000 30 1269 1281 10.1016/S0020-7519(00)00136-3 11113254

[B3] Colwell DD Dantas-Torres F Otranto D Vector-borne parasitic zoonoses: Emerging scenarios and new perspectives Vet Parasitol 2011 182 14 21 10.1016/j.vetpar.2011.07.012 21852040

[B4] Cortes S Vaz Y Neves R Maia C Cardoso L Campino: **Risk factors for canine leishmaniasis in an endemic Mediterranean region** Vet Parasitol 2012 189 189 96 10.1016/j.vetpar.2012.04.028 22575278

[B5] Cardoso L Lopes AP Sherry K Schallig H Solano-Gallego L Low seroprevalence of *Leishmania infantum* infection in cats from northern Portugal based on DAT and ELISA Vet Parasitol 2010 174 37 42 10.1016/j.vetpar.2010.08.022 20851524

[B6] WHO The vector-borne human infections of Europe. Their distribution and burden on Public Health 2004 27 30 Available at: http://www.euro.who.int/__data/assets/pdf_file/0008/98765/e82481.pdf

[B7] Campino L Maia C Epidemiologia das leishmanioses em Portugal Acta Med Port 2010 23 859 864 21144327

[B8] Center for Food Security and Public Health, Institute for International Cooperation in Animal Biologics & OIE: Leishmaniasis (cutaneous and visceral) 2009 2 3 Available at: http://www.bibliotecadigital.ufmg.br/dspace/handle/1843/360/browse?value=Hessem+Miranda+Neiva&type=author

[B9] Neiva H Frequência de anticorpos de Leishmania sp. em Rattus norvegicus no município de Belo Horizonte, Minas Gerais. 2005 2005 Brasil: Dissertação de Mestrado em epidemiologia. Escola de Veterinária – Universidade Federal de Minas Gerais 18 23 Available at: http://dspace.lcc.ufmg.br/dspace/bitstream/1843/BUOS-8BYHAH/1/disserta__o_de_mestrado_de_hessem_miranda_neiva.pdf

[B10] Melo L Detecção de Leishmania sp. em pequenos mamíferos silvestres e sinantrópicos no município de Belo Horizonte, MG 2008 Dissertação de Pós-graduação em Ciências da Saúde. Centro de pesquisas René Rachou, Fundação Oswaldo Cruz 44 72 available at: http://netra.cpqrr.fiocruz.br/download/Dissertacao_Lutiana_Amaral_de_Melo.pdf

[B11] Júnior J Infecção natural por Leishmania spp. em pequenos mamíferos silvestres e sinantrópicos envolvidos na manutenção da leishmaniose tegumentar americana em área endémica da Zona da Mata Norte de Pernambuco, Brasil 2010 Dissertação de Mestrado em Saúde Pública. Fundação Oswaldo Cruz 36 56 Available at: http://www.cpqam.fiocruz.br/bibpdf/2010marinhojunior-jf.pdf

[B12] Oliveira F Pirmez C Pires M Brazil R Pacheco R PCR-based diagnosis for detection of *Leishmania* in skin and blood of rodents from an endemic area of cutaneous and visceral leishmaniasis in Brazil Vet Parasitol 2005 129 219 227 10.1016/j.vetpar.2005.01.005 15845276

[B13] Zulueta A Villarroel E Rodriguez N Feliciangeli M Mazarri M Reyes O Rodriguez V Centeno M Barrios R Ulrich M Epidemiological aspects of american visceral leishmaniasis in an endemic focus in eastern Venezuela Am Trop Med Hyg 1999 61 945 950 10.4269/ajtmh.1999.61.945 10674675

[B14] De Lima H De Guglielmo Z Rodríguez A Convit J Rodriguez N Cotton rats (*Sigmodon hispidus*) and black rats (*Rattus rattus*) as possible reservoirs of *Leishmania* spp. in Lara state, Venezuela Mem Inst Oswaldo Cruz, Rio de Janeiro 2002 97 169 174 10.1590/S0074-02762002000200004 12016437

[B15] Canto-Lara S Wynsberghe N Vargas-González A Ojeda-Farfán F Andrade-Narváez F Use of monoclonal antibodies for the identification of *Leishmania* spp. isolated from humans and wild rodents in the State of Campeche, México Mem Inst Oswaldo Cruz, Rio de Janeiro 1999 94 305 309 10.1590/s0074-02761999000300005 10348978

[B16] Di Bella C Vitale F Russo G Greco A Milazzo C Aloise G Cagnin M Are rodents a potencial reservoir for *Leishmania infantum* in Italy? J M Eco 2003 7 Suppl 125 129

[B17] Psaroulaki A Antoniou M Toumazos P Mazeris A Loannou I Chochlakis D Christophi N Loukaides P Patsias A Moschandrea I Tselentis Y Rats as indicators of the presence and dispersal of six zoonotic microbial agents in Cyprus, an island ecosystem: a seroepidemiological study Trans R Soc Trop Med Hyg 2010 104 733 739 10.1016/j.trstmh.2010.08.005 20870259

[B18] Papadogiannakis E Spanakos G Kontos V Menounos PG Tegos N Vakalis N Molecular detection of *Leishmania infantum* in wild rodents (*Rattus norvegicus*) in Greece Zoonoses Public Health 2010 57 e23 e25 10.1111/j.1863-2378.2009.01264.x 19912600

[B19] Mohebali M Javadian E Yaghoobi-Ershadi M Akhavan AA Hajjaran H Abaei MR Characterization of *Leishmania* infection in rodents from endemic areas of the Islamic Republic of Iran East Mediterr Health J 2004 10 591 9 16335651

[B20] Pourhohammadi B Motazedian MH Kalantari M Rodent infection with *Leishmania* in a new focus of human cutaneous leishmaniasis, in northern Iran Ann Trop Med Parasitol 2008 102 127 133 10.1179/136485908X252223 18318934

[B21] Akhavan A Mirhendi H Khamesipour A Alimohammadian M Rassi Y Bates P Kamhawi S Valenzuela J Arandian M Abdoli H Jalali-zand N Jafari R Shareghi N Ghanei M Yaghoobi-Ershadi M *Leishmania* species: Detection and identification by nested PCR assay from skin samples of rodent reservoirs Exp Parasitol 2010 126 552 556 10.1016/j.exppara.2010.06.003 20566364 PMC2939322

[B22] Motazedian M Parhizkari M Mehrabani D Hatam G Asgari Q First detection of *Leishmania major* in *Rattus norvegicus* from Fars Province, Southern Iran Vector Borne Zoonotic Dis 2010 10 969 75 10.1089/vbz.2008.0214 20426685

[B23] Parhizkari M Motazedian MH Asgari Q Mehrabani D The PCR-based detection of *Leishmania major* in *Mus musculus* and other rodents caught in Southern Iran: A guide to sample selection Ann Trop Med Parasitol 2011 105 319 323 10.1179/136485911X12987676649827 21871168 PMC4090797

[B24] Ministério da Saúde Manual de Controle de Roedores - vigilância Epidemiológica 2002 Brasília: Funasa 20 21 Available at: http://portal.saude.gov.br/portal/arquivos/pdf/manual_roedores.pdf

[B25] Ready PD Leishmaniasis emergence in Europe Euro Surveill 2010 15 3 4 Available at: http://eurosurveillance.org/images/dynamic/EE/V15N10/art19505.pdf

[B26] Svobodová M Votýpka J Nicolas L Volf P *Leishmania tropica* in the black rat (*Rattus rattus*): persistence and transmission from asymptomatic host to sand fly vector *Phlebotomus sergenti* Microbes Infect 2003 5 361 364 10.1016/S1286-4579(03)00046-7 12737990

